# Prognostic Value of Masseter Muscle Index, Masseter Radiodensity, and the Global Immune-Nutrition-Inflammation Index in Laryngeal Carcinoma Treated with Definitive Radiotherapy

**DOI:** 10.3390/cancers18182981

**Published:** 2026-09-15

**Authors:** Mehmet Kızılkaya, Timur Koca, Aylin Fidan Korcum

**Affiliations:** 1 Department of Radiation Oncology, Trabzon Kanuni Training and Research Hospital, 61250 Trabzon, Türkiye; 2 Department of Radiation Oncology, Akdeniz University, 07070 Antalya, Türkiye; timurkoca@akdeniz.edu.tr (T.K.); aylinkorcum@akdeniz.edu.tr (A.F.K.)

**Keywords:** myopenia, myosteatosis, Global Immune–Nutrition–Inflammation Index, masseter muscle index, laryngeal cancer

## Abstract

Background: This retrospective study evaluated pretreatment masseter muscle index (MMI), masseter radiodensity (HUAC), and Global Immune–Nutrition–Inflammation Index (GINI) as prognostic markers in patients with laryngeal squamous cell carcinoma treated with definitive radiotherapy. Methods: MMI and HUAC were measured on simulation computed tomography, and GINI was calculated from pretreatment laboratory values. Biomarkers were analyzed continuously using Firth penalized Cox models adjusted for age, performance status, tumor site, and T and N categories. Nonlinearity was assessed using restricted cubic splines. Joint models and multiple imputation assessed robustness. HUAC associations were assessed over time. Exploratory cutoffs were derived using censoring-aware three-year time-dependent receiver operating characteristic analysis, with radiotherapy initiation as time zero. Results: Among 146 patients, 30 died and 48 experienced progression or death; GINI was available in 116. Each standard-deviation decrease in MMI was associated with worse overall survival (OS; adjusted hazard ratio 1.92, 95% confidence interval 1.21–3.05; *p* = 0.002) and progression-free survival (PFS; 1.91, 1.35–2.71; *p* < 0.001). These associations weakened after further adjustment for body mass index and concurrent systemic therapy. GINI findings were not robust in adjusted, joint, and missing-data analyses. Lower HUAC showed an exploratory association with poorer outcomes later in follow-up, based on few late events. Three-year OS/PFS estimates were 92.5%/81.1% versus 68.9%/59.5% for MMI > 2.830 versus ≤2.830 cm^2^/m^2^, and 90.2%/81.9% versus 45.1%/38.0% for GINI < 139.67 versus ≥139.67. The GINI cutoff was unstable, with a 95% bootstrap interval of 20.104–180.985. Conclusions: Lower MMI showed the most consistent prognostic association. This initial evaluation of GINI in laryngeal cancer supports further investigation of its prognostic potential. HUAC findings and cohort-derived cutoffs remain exploratory and require external validation.

## Simple Summary

Patients with laryngeal squamous cell carcinoma may experience different outcomes despite similar disease stage. We evaluated pretreatment masseter muscle index (MMI), masseter radiodensity (HUAC), and the Global Immune–Nutrition–Inflammation Index (GINI) in 146 patients treated with definitive radiotherapy; GINI was available in 116 patients. Lower MMI showed the most consistent adverse prognostic association. Higher GINI was associated with poorer outcomes in univariable and exploratory threshold-based analyses, but its association weakened after event-conscious multivariable adjustment, joint modeling with MMI, and missing-data sensitivity analysis. HUAC showed no statistically significant association in constant-effect models; exploratory extended Cox analyses suggested that the association of lower HUAC with adverse outcomes increased over time, although late estimates were based on few events. These routinely available measurements may support risk assessment, but the findings and cohort-specific thresholds require external validation and should not currently guide treatment decisions. Using the exploratory cohort-specific thresholds, three-year OS/PFS were 92.5%/81.1% versus 68.9%/59.5% for MMI > 2.830 versus ≤2.830 cm^2^/m^2^, and 90.2%/81.9% versus 45.1%/38.0% for GINI < 139.67 versus ≥139.67.

## 1. Introduction

Larynx cancer is one of the common subtypes of head and neck squamous cell carcinoma. According to GLOBOCAN 2022 estimates, approximately 189,000 new cases of laryngeal cancer and 103,000 related deaths occurred worldwide in 2022 [1]. Tobacco use and alcohol consumption are the major risk factors for laryngeal cancer [2]. Although the five-year survival rate can exceed 80% in early-stage laryngeal cancer, it declines markedly in patients with advanced-stage disease [3]. Prognosis is influenced by several factors, most notably disease stage, as well as the anatomical subsite of the primary tumor, degree of differentiation, performance status, and age [4].

Identifying patients at an increased risk of poor outcomes is important for individualizing treatment and follow-up strategies. In recent years, research has increasingly focused on biomarkers that can predict cancer progression and survival outcomes. In particular, systemic inflammatory biomarkers and computed tomography (CT)-derived measures of muscle quantity and radiodensity have received increasing attention.

Sarcopenia is formally characterized by reductions in muscle strength and physical performance, in addition to low muscle quantity. Nevertheless, in retrospective oncology studies without functional assessments, the term sarcopenia is frequently used to describe CT-defined low muscle quantity or reduced muscle radiodensity [5]. Low muscle quantity is discussed in relation to myopenia, whereas reduced muscle radiodensity is used as an indirect imaging indicator of myosteatosis [5,6]. The masseter muscle has increasingly been used to assess myopenia and myosteatosis in oncological studies. Because it is routinely included within the field of view of head and neck imaging, it can be measured without requiring an additional imaging protocol. Masseter muscle–based measures of myopenia and myosteatosis have been reported to be associated with adverse survival outcomes in several malignancies, including nasopharyngeal cancer, glioblastoma, esophageal cancer, and other head and neck cancers [7,8,9,10].

A growing body of evidence suggests that systemic inflammation may play an important role in cancer development, progression, and prognosis [11]. The prognostic significance of various inflammatory biomarkers has been investigated in patients with laryngeal cancer, and several of these biomarkers, whether assessed individually or in combination, have been associated with the clinical outcomes. The Global Immune–Nutrition–Inflammation Index (GINI) is a comprehensive composite biomarker that integrates immunological, inflammatory, and nutritional parameters [12]. It is calculated using pretreatment C-reactive protein (CRP), albumin, monocyte, platelet, neutrophil, and lymphocyte measurements, all of which are readily available from routine hematological and biochemical tests without requiring additional testing. The GINI was first introduced by Topkan et al. in patients with stage IIIC non-small cell lung cancer and was found to be significantly associated with survival outcomes [12]. It has subsequently been investigated in other malignancies, including glioblastoma and esophageal cancer, where its prognostic value has been demonstrated [13,14]. However, its prognostic significance in laryngeal cancer remains unknown.

To our knowledge, no previous study has evaluated masseter muscle index (MMI), masseter radiodensity (HUAC), and GINI within the same cohort of patients with laryngeal cancer treated with definitive radiotherapy. We therefore evaluated their associations with overall survival (OS) and progression-free survival (PFS), treating the biomarkers primarily as continuous variables and categorical thresholds as exploratory, cohort-specific risk-group definitions.

## 2. Materials and Methods

### 2.1. Study Design and Patient Selection

We retrospectively reviewed patients who received definitive radiotherapy for laryngeal squamous cell carcinoma at the Department of Radiation Oncology, Akdeniz University Faculty of Medicine, between 1 January 2015 and 30 June 2024. The study was approved by the Akdeniz University Faculty of Medicine Clinical Research Ethics Committee on 6 March 2025 (decision no. 232). Reporting was guided by the Strengthening the Reporting of Observational Studies in Epidemiology (STROBE) statement [15].

**Figure 1:**
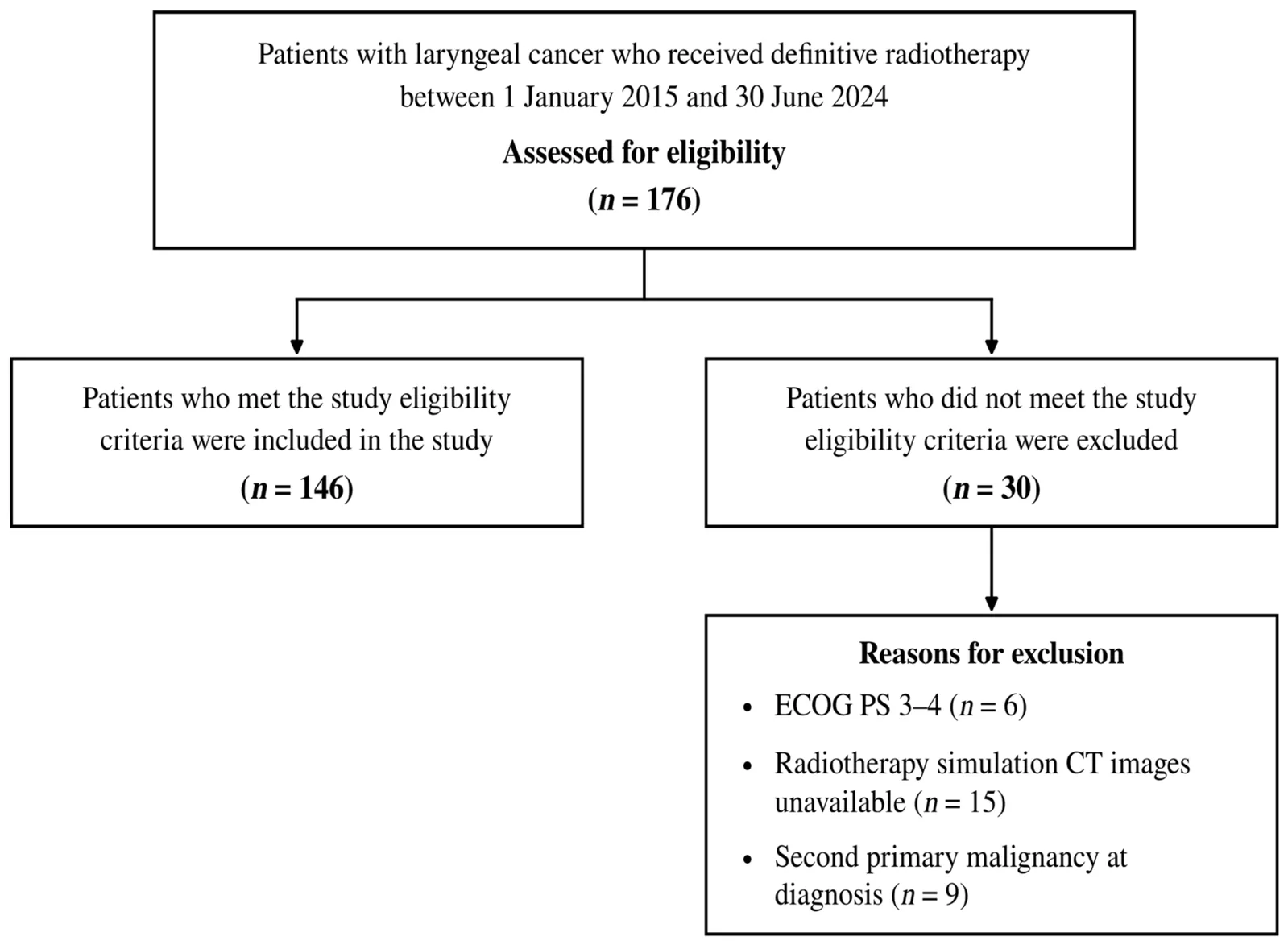
Patient-selection flow diagram.

A total of 176 patients were assessed for eligibility. Thirty were excluded: 9 because of another primary malignancy, 6 because of Eastern Cooperative Oncology Group performance status (ECOG PS) 3–4, and 15 because radiotherapy simulation CT images were unavailable. The final cohort comprised 146 patients (Figure 1). Patients with ECOG PS 3–4 were excluded because severe baseline functional impairment may influence treatment tolerance, treatment completion, nutritional status, and survival. This restriction was intended to reduce clinical heterogeneity related to baseline functional status. The cohort was therefore restricted to patients with ECOG PS 0–2; consequently, the findings may not be generalizable to patients with ECOG PS 3–4.

### 2.2. Treatment and Follow-Up

Definitive radiotherapy was delivered to total doses of 63–70 Gy using intensity-modulated radiotherapy (IMRT) in 131 patients (89.7%) and three-dimensional conformal radiotherapy (3D-CRT) in 15 patients (10.3%). Fraction sizes in the dataset were 1.80 Gy (*n* = 1), 2.00 Gy (*n* = 76), 2.12 Gy (*n* = 6), and 2.25 Gy (*n* = 63). All 146 patients completed the planned radiotherapy course. The elapsed time from the first to the final fraction was a median of 48 days (IQR 42–51; range 37–59). Dates and reasons for individual unscheduled treatment interruptions were not separately coded. Concurrent systemic therapy was administered to 69 patients (47.3%): 57 received cisplatin and 12 received cetuximab. Cisplatin was administered weekly at 40 mg/m^2^, with a planned cumulative dose exceeding 200 mg/m^2^. Patients considered unsuitable for cisplatin-based chemoradiotherapy received concurrent cetuximab: a 400 mg/m^2^ loading dose one week before radiotherapy, followed by 250 mg/m^2^ once weekly throughout radiotherapy.

After the completion of primary treatment, patients were generally assessed every 3–6 months for the first 2 years and every 6–12 months for the subsequent 3 years. Follow-up evaluations included physical examination, laryngoscopy, magnetic resonance imaging, and/or positron emission tomography/CT as clinically indicated.

### 2.3. Clinical Data and Staging

Demographic, clinical, pathological, and treatment data were obtained from hospital records, including age, sex, height, weight, ECOG PS, smoking history, tumor characteristics, complete blood count, CRP level, and albumin level. Clinical staging was based on physical examination and available CT, magnetic resonance imaging, and positron emission tomography/CT findings and was assigned according to the eighth edition of the American Joint Committee on Cancer TNM classification.

### 2.4. Masseter Muscle Measurements

**Figure 2:**
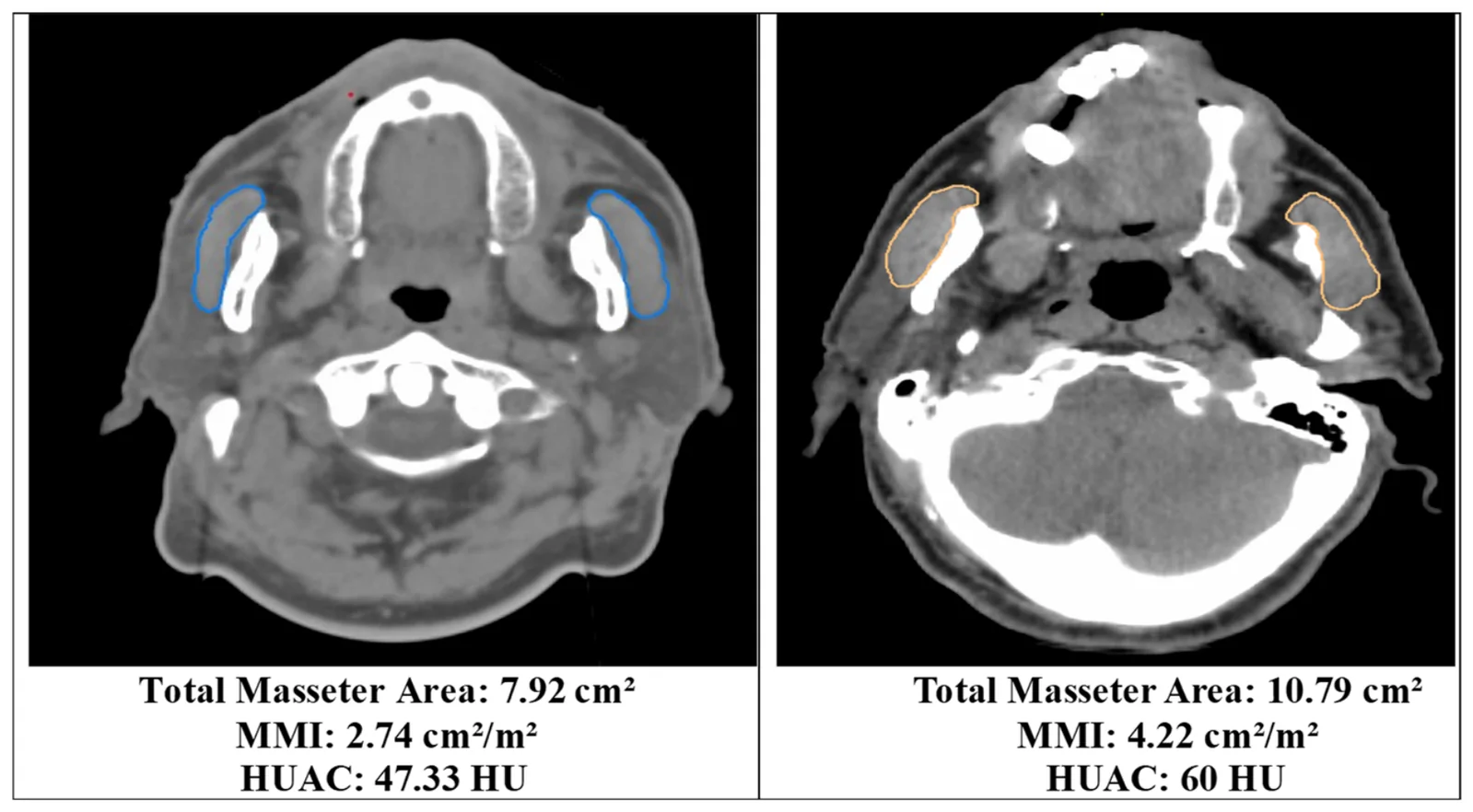
Representative axial CT images showing bilateral masseter delineation and corresponding MMI and HUAC measurements. The left example has a total masseter area of 7.92 cm^2^, an MMI of 2.74 cm^2^/m^2^, and an HUAC of 47.33 HU. The corresponding values in the right example are 10.79 cm^2^, 4.22 cm^2^/m^2^, and 60 HU, respectively. These examples illustrate differences in both masseter muscle quantity and radiodensity.

MMI and HUAC were derived from bilateral masseter measurements on one axial slice 2 cm below the zygomatic arch [9,10,16] (Figure 2). Pretreatment radiotherapy simulation CT images were acquired without intravenous contrast and reconstructed using the standard reconstruction kernel (Stnd; GE HealthCare, Chicago, IL, USA), with a 2.5 mm slice thickness. Removable dentures were removed before CT simulation, and no dental artifacts affecting the masseter measurements were observed. Image analysis was performed using Monaco (Elekta, Stockholm, Sweden). Both masseter muscles were initially segmented manually by a radiation oncologist with 6 years of experience. The contours were subsequently reviewed with a second radiation oncologist with more than 20 years of experience, and the final contours used for analysis were agreed upon by consensus. Only voxels within −29 to +150 HU were retained for both cross-sectional area and attenuation calculations. HUAC was calculated as the area-weighted mean attenuation of the retained voxels in the bilateral masseter muscles. Independent repeat measurements were not performed; therefore, interobserver and intraobserver reproducibility could not be quantified using intraclass correlation coefficients (ICCs) or Bland–Altman agreement plots.

MMI was calculated by dividing the sum of the right and left masseter cross-sectional areas (cm^2^) by height in meters squared. HUAC was calculated as the area-weighted mean attenuation of the bilateral masseter muscles.

MMI = (RMA + LMA)/height^2^

HUAC = (RMA × RMD + LMA × LMD)/(RMA + LMA)

RMA and LMA denote the right and left masseter areas (cm^2^), respectively, and RMD and LMD denote the corresponding mean attenuation values (HU). MMI was expressed as cm^2^/m^2^, and HUAC as HU.

### 2.5. Global Immune–Nutrition–Inflammation Index

GINI was calculated from the pretreatment laboratory measurements closest to radiotherapy initiation. Among the 116 patients with calculable GINI, the median interval between blood collection and radiotherapy initiation was 2 days (IQR 1–5.25; range 0–24); an interval of 0 days denotes sampling on the date of radiotherapy initiation. At the time of blood sampling, patients had no acute infection or active inflammatory condition, were not using corticosteroids, and had not undergone recent surgery. Recent biopsies and other invasive procedures were also considered when selecting laboratory measurements. CRP was expressed in mg/L, albumin in g/L, and absolute monocyte, platelet, neutrophil, and lymphocyte counts in ×10^9^/L, numerically equivalent to ×10^3^/µL.

The components of GINI represent complementary inflammatory, immune, and nutritional domains. CRP reflects the systemic acute-phase inflammatory response, whereas albumin reflects both nutritional and inflammatory status. Neutrophils and monocytes represent innate inflammatory and myeloid activity, platelets are associated with tumor-related inflammation and angiogenesis, and lymphocytes reflect adaptive antitumor immune activity. Hemoglobin and other nutritional markers may also have prognostic relevance; however, they are not components of the originally developed GINI formula. Incorporating these variables into the GINI formula would constitute a modification of the established index and would require separate evaluation and validation. They could also be evaluated separately as additional prognostic markers without altering GINI; however, their incremental prognostic value was not assessed in the present study. Therefore, GINI was calculated according to its original definition in the present study.

$$\mathrm{GINI}=\frac{\mathrm{CRP}\;\times \;\mathrm{Monocyte}\;\mathrm{count}\;\times \;\mathrm{Platelet}\;\mathrm{count}\;\times \;\mathrm{Neutrophil}\;\mathrm{count}}{\mathrm{Albumin}\;\times \;\mathrm{Lymphocyte}\;\mathrm{count}}$$

CRP was recorded in mg/L, albumin in g/L, and absolute monocyte, platelet, neutrophil, and lymphocyte counts in ×10^9^/L.

GINI was available in 116 patients. Pretreatment albumin was the only unavailable component required to calculate GINI in 30 patients. Albumin results were available only after radiotherapy initiation in 16 patients, whereas no albumin results could be retrieved for the remaining 14 patients. Post-treatment-initiation albumin measurements were not used to calculate pretreatment GINI. Complete-case and multiple-imputation sensitivity analyses were therefore performed, and patients with and without calculable GINI were compared for baseline characteristics, treatment, follow-up, and outcomes.

### 2.6. Survival Endpoints

OS and PFS were measured from radiotherapy initiation, which was selected as the common time origin because the imaging and laboratory biomarkers were obtained before treatment. OS was defined as time to death from any cause; patients alive at last follow-up were censored on that date. PFS was defined as time to first documented progression or death from any cause, whichever occurred first. The progression date was the earliest recorded date of local or regional recurrence, or distant metastasis. Patients without progression or death were censored at their last documented follow-up. The diagnosis-to-radiotherapy interval was summarized descriptively.

### 2.7. Statistical Analysis

Statistical analyses were performed using IBM SPSS Statistics (version 24.0; IBM Corp., Armonk, NY, USA) and Python (version 3.12), including the firthmodels package (version 0.8.2) for Firth penalized Cox regression. All tests were two-sided, and statistical significance was set at *p* < 0.05. Continuous variables were summarized using medians and interquartile ranges, and categorical variables using counts and percentages. MMI and HUAC were modeled per 1-standard-deviation decrease; GINI was log2-transformed and modeled per doubling. Nonlinearity was assessed using restricted cubic splines with three knots at the 10th, 50th, and 90th percentiles of each continuous marker. Univariable spline and linear Cox models were compared using a one-degree-of-freedom likelihood-ratio test. Given the univariable HUAC nonlinearity signal, sensitivity analyses additionally assessed nonlinearity after adjustment for the core confounders and by adding the nonlinear spline term and its log-time interaction to the adjusted extended Cox model. Because the number of events was limited, Firth penalized Cox regression was used with clinically selected core confounders: age (continuous), ECOG PS ≥2, non-glottic primary site, T3–4 category, and N-positive disease. Expanded sensitivity models additionally included body mass index (BMI) as a continuous variable and concurrent systemic therapy as a binary variable (yes/no). Joint MMI–GINI models were fitted in the 116 patients with both measurements using both the core and expanded adjustment sets. The smaller core models were designated as primary because of the limited number of events; the expanded models assessed sensitivity to additional adjustment and were not intended to estimate causal treatment effects.

GINI missingness was assessed by comparing patients with and without calculable GINI. Fifty imputed datasets were generated for missing albumin using bootstrap-based predictive mean matching with five nearest donors. Predictors included age, BMI, MMI, HUAC, the available GINI laboratory components, ECOG performance status, primary site, T category, N status, concurrent systemic therapy, treatment year, and event indicators and Nelson–Aalen cumulative hazard estimates for OS and PFS. Survival times were calculated from radiotherapy initiation. GINI was recalculated within each completed dataset, and Firth penalized Cox models used the expanded adjustment set. Coefficients and variances were pooled using Rubin’s rules. This sensitivity analysis assumed that albumin values were missing at random conditional on the included variables. Proportional hazards were assessed using scaled Schoenfeld residuals with a rank transformation of time in corresponding ordinary Cox models containing the same core covariates. Because the HUAC covariate-specific tests indicated non-proportionality, an extended Cox model included HUAC and a HUAC × log(time/36 months) interaction, adjusted for the same core confounders. Time-specific HUAC hazard ratios (HRs) were estimated at 12, 24, 36, and 60 months. A complementary piecewise extended Cox analysis estimated separate HUAC effects during 0–36 and >36 months; the 36-month boundary matched the clinically defined ROC horizon.

Categorical analyses were secondary and exploratory. Cumulative/dynamic time-dependent ROC analysis at 36 months from radiotherapy initiation used inverse probability of censoring weighting, with the censoring distribution estimated by reverse Kaplan–Meier analysis. The 36-month horizon was selected for the revised analysis because median follow-up was approximately 49 months and follow-up support was substantially lower at five years. Lower MMI and HUAC and higher GINI were specified as the adverse directions. Thresholds maximizing the Youden index were represented by midpoints between adjacent observed values; tied maxima were resolved by selecting the smallest high-risk group. Patient-level bootstrap resampling with 1000 replicates re-estimated the censoring distribution and reselected the threshold in each replicate. Percentile intervals were calculated for the AUCs and thresholds. Optimism in Harrell’s C-index for the cutoff-based grouping was estimated as the mean difference between bootstrap-sample and original-sample performance and subtracted from the apparent C-index. The OS-derived MMI and GINI thresholds were used for both OS and PFS visualization. Kaplan–Meier estimates were accompanied by pointwise 95% confidence intervals using Greenwood’s variance and a log-log transformation, confidence bands, and numbers at risk. These intervals and log-rank tests were conditional on the selected thresholds and were interpreted as exploratory. All tests were two-sided; *p* < 0.05 was considered statistically significant.

## 3. Results

### 3.1. Patient Characteristics

A total of 146 patients were included (Table 1). At the end of follow-up, 116 patients were alive and 30 had died. The 48 PFS events comprised 31 documented progressions and 17 deaths without previous documented progression; 98 patients were censored for PFS. The median diagnosis-to-radiotherapy interval was 25 days (IQR 16–37; range 0–59). GINI was calculable in 116 patients; the 30 patients without GINI had 2 deaths and 8 PFS events, compared with 28 deaths and 40 PFS events among complete cases. Missing-GINI patients more often had glottic, earlier-stage disease and less often received concurrent systemic therapy, indicating a material risk of selection bias (Table S1).

### 3.2. Exploratory Three-Year Time-Dependent ROC Analysis

**Figure 3:**
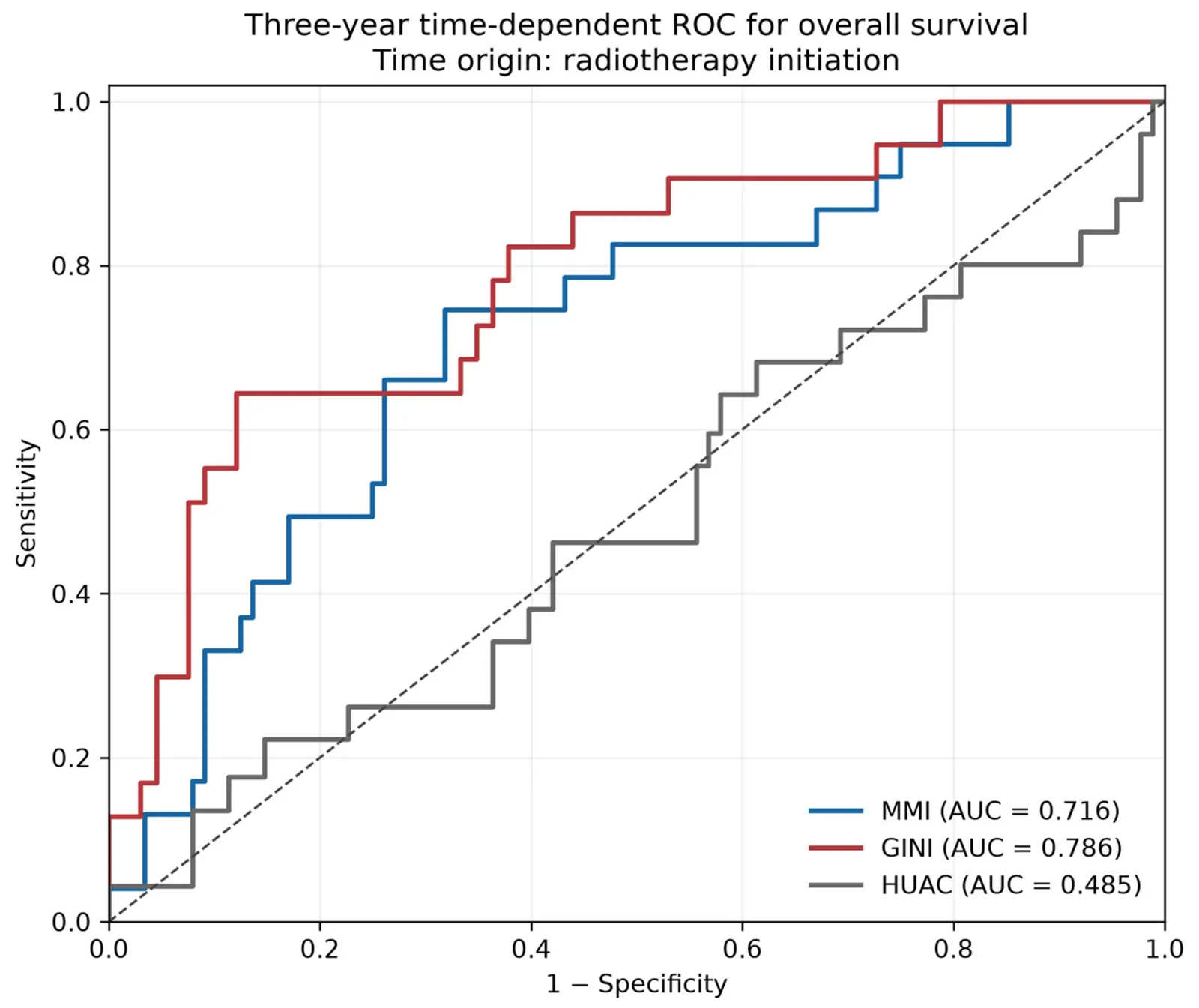
Cumulative/dynamic time-dependent ROC curves for three-year overall survival from radiotherapy initiation. AUCs were estimated using inverse probability of censoring weighting. Lower MMI/HUAC and higher GINI were specified as adverse directions. The diagonal dashed line represents chance-level discrimination (AUC = 0.50).

At 36 months from radiotherapy initiation, the exploratory cohort-specific cutoffs were 2.830 cm^2^/m^2^ for MMI and 139.67 for GINI. For MMI, the AUC was 0.716 (95% bootstrap CI 0.592–0.833), with sensitivity 74.6% and specificity 68.2%. For GINI, the AUC was 0.786 (95% bootstrap CI 0.666–0.892), with sensitivity 64.4% and specificity 87.9%. The 95% bootstrap cutoff intervals were 2.433–3.027 for MMI and 20.104–180.985 for GINI. HUAC showed no useful discrimination in the specified adverse direction (AUC 0.485, 95% bootstrap CI 0.344–0.621) and was not retained for threshold-based survival visualization. All thresholds were considered exploratory and cohort-specific (Table 2; Figure 3).

### 3.3. Survival Analysis

The median follow-up estimated by reverse Kaplan–Meier analysis from radiotherapy initiation was 49.3 months. At 36 and 60 months, 88 and 37 patients, respectively, remained at risk for OS. Exploratory Kaplan–Meier analyses used the three-year OS-derived thresholds for both endpoints; no separate PFS thresholds were selected.

**Figure 4:**
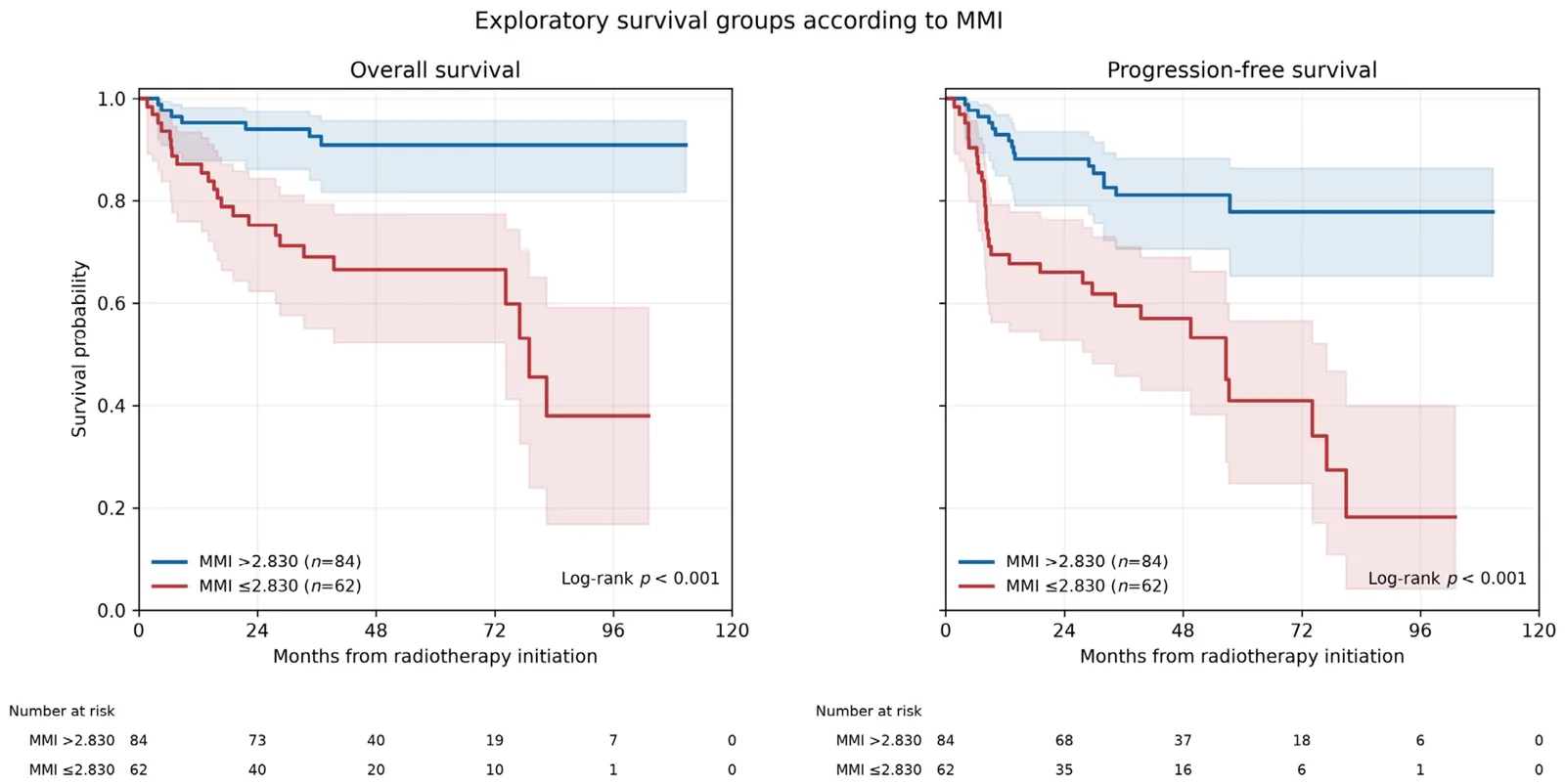
Exploratory OS and PFS curves according to MMI > 2.830 versus ≤2.830 cm^2^/m^2^. Time was measured from radiotherapy initiation. Pointwise 95% confidence bands and numbers at risk are shown.

For MMI > 2.830 versus ≤2.830 cm^2^/m^2^, three-year OS was 92.5% (95% CI 84.0–96.6) versus 68.9% (95% CI 55.1–79.3), and three-year PFS was 81.1% (95% CI 70.5–88.2) versus 59.5% (95% CI 45.6–70.9); both log-rank *p* < 0.001 (Table 3; Figure 4). These categorical findings are exploratory and do not establish a validated definition of myopenia.

**Figure 5:**
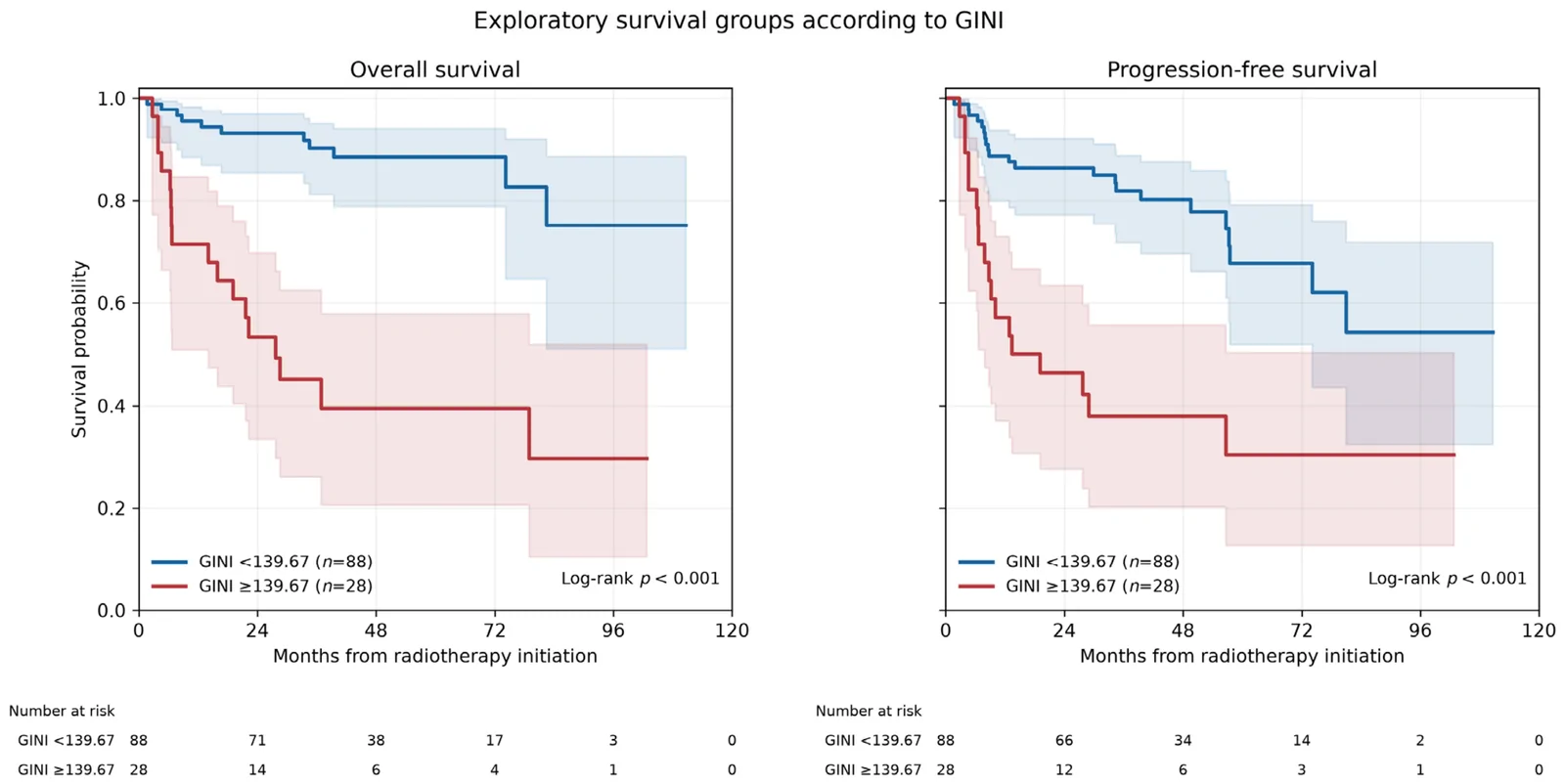
Exploratory OS and PFS curves according to GINI < 139.67 versus ≥139.67. Time was measured from radiotherapy initiation. Pointwise 95% confidence bands and numbers at risk are shown.

For GINI < 139.67 versus ≥139.67, three-year OS was 90.2% (95% CI 81.2–95.0) versus 45.1% (95% CI 26.0–62.5), and three-year PFS was 81.9% (95% CI 71.6–88.7) versus 38.0% (95% CI 20.2–55.7); both log-rank *p* < 0.001 (Table 4; Figure 5). The broad bootstrap interval indicates substantial uncertainty in this exploratory threshold.

### 3.4. Cox Regression Analyses

In continuous univariable analyses, each 1-SD decrease in MMI was associated with OS (HR 2.30, 95% CI 1.48–3.60; *p* < 0.001) and PFS (HR 2.14, 95% CI 1.53–3.01; *p* < 0.001; Table 5). Each doubling of GINI was associated with OS (HR 1.42, 95% CI 1.23–1.64; *p* < 0.001) and PFS (HR 1.24, 95% CI 1.10–1.40; *p* < 0.001; Table 6). Linear univariable HUAC terms were not statistically significant for OS or PFS. Restricted cubic spline analyses showed no evidence of nonlinearity for MMI (OS *p* = 0.708; PFS *p* = 0.948) or log2 GINI (OS *p* = 0.452; PFS *p* = 0.501). HUAC showed an univariable nonlinearity signal for PFS (*p* = 0.039; OS *p* = 0.054). This was not statistically significant after adjustment for the core confounders (OS *p* = 0.232; PFS *p* = 0.099). Adding nonlinear HUAC and nonlinear HUAC-by-log-time terms to the adjusted extended Cox model did not significantly improve fit (OS *p* = 0.957; PFS *p* = 0.387; Table S2).

The 116 complete cases accounted for 28 deaths and 40 PFS events. The unequal clinical composition and outcome distribution between patients with and without GINI supported additional missing-data sensitivity analyses rather than reliance on complete cases alone.

In Firth penalized models adjusted for age, ECOG PS, primary site, T category, and N status, a 1-SD decrease in MMI remained associated with OS (aHR 1.92, 95% CI 1.21–3.05; *p* = 0.002) and PFS (aHR 1.91, 95% CI 1.35–2.71; *p* < 0.001). In these constant-effect models, HUAC was not significantly associated with either endpoint. Per doubling of GINI, the adjusted HR was 1.19 (95% CI 0.99–1.41; *p* = 0.050) for OS and 1.08 (95% CI 0.93–1.24; *p* = 0.309) for PFS (Table 7A).

In the joint MMI–GINI model adjusted for the core covariates, MMI was associated with OS (aHR 1.81, 95% CI 1.15–2.86; *p* = 0.004) and PFS (aHR 1.78, 95% CI 1.23–2.58; *p* < 0.001), whereas GINI did not show a statistically significant independent association with either endpoint (OS *p* = 0.061; PFS *p* = 0.369; Table 7B). In the expanded single-marker models, the MMI association was attenuated for OS but remained statistically significant for PFS. In the expanded joint MMI–GINI model, the MMI estimates were attenuated for both OS (aHR 1.64, 95% CI 0.91–2.95; *p* = 0.086) and PFS (aHR 1.56, 95% CI 0.98–2.46; *p* = 0.051). GINI remained statistically nonsignificant in the expanded joint model (OS *p* = 0.083; PFS *p* = 0.395; Table 7C). After multiple imputation of missing albumin, GINI showed no statistically significant adjusted association with OS (HR per doubling 1.15, 95% CI 0.97–1.37; *p* = 0.112) or PFS (HR per doubling 1.03, 95% CI 0.90–1.18; *p* = 0.647; Table 7C). Scaled Schoenfeld residual tests in the core-adjusted Cox models indicated non-proportional hazards for HUAC (OS *p* < 0.001; PFS *p* = 0.004). In adjusted extended Cox models, the effect of a 1-SD decrease in HUAC increased with log time (interaction *p* < 0.001 for OS and *p* = 0.002 for PFS). At 12, 36, and 60 months, estimated HRs were respectively 0.90 (95% CI 0.57–1.41), 2.23 (1.18–4.21), and 3.41 (1.53–7.62) for OS, and 0.98 (0.69–1.37), 1.71 (1.04–2.79), and 2.22 (1.19–4.12) for PFS. In the complementary 36-month piecewise model, lower HUAC showed no statistically significant association during 0–36 months but was associated with higher hazards after 36 months. Only 6 OS and 9 PFS events occurred after 36 months; therefore, the late estimates were interpreted as exploratory and imprecise (Table 8).

## 4. Discussion

The present analyses identify lower MMI as the most consistent prognostic marker. GINI showed strong univariable and exploratory categorical associations, but these were attenuated after event-conscious penalized adjustment, joint modeling with MMI, and multiple imputation. HUAC showed no statistically significant association in constant-effect models, but extended Cox analyses suggested that lower HUAC became adverse later in follow-up. The findings do not support describing MMI and GINI as providing independently complementary information.

Exploratory categorical analyses showed absolute differences in survival estimates between the threshold-defined groups. Three-year OS and PFS were 92.5% and 81.1%, respectively, in patients with MMI > 2.830 cm^2^/m^2^, compared with 68.9% and 59.5% in those with MMI ≤ 2.830 cm^2^/m^2^. Corresponding estimates were 90.2% and 81.9% for GINI < 139.67, compared with 45.1% and 38.0% for GINI ≥ 139.67. These findings remain exploratory because the thresholds were selected and evaluated in the same cohort. Bootstrap correction of threshold-based discrimination does not externally validate the thresholds or eliminate optimism in the group-specific survival estimates.

Several previous studies have used the masseter muscle to assess myopenia. McGoldrick et al. demonstrated that myopenia, defined according to the masseter muscle area (MMA), was associated with shorter overall survival (OS) in patients aged ≥65 years with head and neck cancer [10]. In a study involving 99 patients with head and neck cancer, van Heusden et al. reported that masseter muscle parameters correlated with skeletal muscle area measured at the C3 and L3 vertebral levels, and that myopenia defined by the masseter muscle index (MMI) was a significant prognostic factor for OS [17]. Pehlivan et al. found that myopenia, defined according to the total masseter muscle volume (MMV), was associated with shorter OS and progression-free survival (PFS) in 97 patients with locally advanced nasopharyngeal cancer [7]. Similar prognostic associations involving the masseter muscle have also been demonstrated in glioblastoma and esophageal cancer [8,9].

Masseter-based assessment is particularly practical in head and neck oncology because the muscle is consistently included in routine radiotherapy simulation CT, allowing body-composition assessment without additional imaging or abdominal coverage. It also has direct functional relevance to mastication and nutritional intake, domains that are frequently compromised in laryngeal cancer. Prior correlations with C3- and L3-derived muscle measures support its use as a feasible regional surrogate [17]. Nevertheless, masseter measurements are not interchangeable with whole-body muscle mass, lumbar or cervical skeletal-muscle indices, or direct strength and performance testing.

In the present study, MMI was evaluated as a continuous measure of regional muscle quantity and an exploratory imaging indicator of myopenia, rather than as a validated diagnostic criterion. Each 1-SD decrease was associated with approximately 1.9-fold higher hazards of OS and PFS events in the core penalized models. The 2.830 cm^2^/m^2^ threshold is reported only as a cohort-specific low-MMI cutoff for exploratory visualization; it is not a validated diagnostic definition of myopenia.

Inflammation promotes tumor cell proliferation, angiogenesis, and metastatic potential within the tumor microenvironment [18]. Recent studies have reported the prognostic value of the neutrophil-to-lymphocyte ratio, platelet-to-lymphocyte ratio, monocyte-to-lymphocyte ratio, pan-immune-inflammation value (PIV), C-reactive protein-to-albumin ratio (CAR), and other composite inflammatory markers in laryngeal cancer and other head and neck cancers [19,20,21]. Collectively, these studies suggest that systemic inflammation and the immune and nutritional status of the host may influence the prognosis of laryngeal cancer. However, the role of the Global Immune–Nutrition–Inflammation Index (GINI) in laryngeal cancer has not yet been established. GINI was first introduced by Topkan et al. and is calculated using the following formula: (CRP × monocyte count × platelet count × neutrophil count)/(albumin × lymphocyte count) [12]. It integrates the systemic inflammatory response and immunonutritional status into a single index. In their study of 802 patients with stage IIIC non-small cell lung cancer treated with definitive chemoradiotherapy, Topkan et al. identified an optimal pretreatment GINI cut-off value of 1562. Patients with a GINI ≥ 1562 had significantly shorter OS, PFS, and LRFS [12]. Significant associations between high GINI values and shorter survival have also been demonstrated in patients with glioblastoma, esophageal cancer, and grade 4 diffuse glioma [13,14,22].

Although the prognostic value of GINI has not been previously investigated in patients with laryngeal cancer, its individual components have been studied. GINI can also be mathematically expressed as the product of CAR and PIV, both of which have been separately evaluated as prognostic markers in laryngeal cancer. For example, Shi et al. demonstrated that high PIV was associated with shorter OS and PFS in patients with laryngeal and pharyngeal cancers and remained an independent adverse prognostic factor in a multivariable analysis [23]. Other studies involving patients with head and neck cancer have similarly identified high PIV as an adverse prognostic factor associated with significantly shorter OS [24,25]. Regarding CAR, Yu et al. evaluated 129 patients with laryngeal squamous cell carcinoma and found that high CAR was significantly associated with nodal metastasis, advanced disease stage, and recurrence and was an independent prognostic factor for both OS and DFS [26]. In another study involving 56 surgically treated patients with laryngeal or pharyngeal cancer, high CAR values were associated with shorter survival, highlighting the potential prognostic relevance of this marker [27].

NLR and PLR are calculated as the neutrophil-to-lymphocyte and platelet-to-lymphocyte ratios, respectively, whereas the prognostic nutritional index (PNI) combines serum albumin and lymphocyte count to reflect nutritional and immune status. GINI integrates a broader spectrum of inflammatory, immune, and nutritional components, including CRP, albumin, neutrophils, lymphocytes, monocytes, and platelets. MMI and HUAC, however, provide CT-derived information on regional muscle quantity and radiodensity and therefore capture a different aspect of the host phenotype. Accordingly, these biomarkers should be regarded as biologically distinct but potentially related measures rather than interchangeable alternatives. Although the broader composition of GINI may theoretically provide a more comprehensive assessment, the present study did not directly demonstrate superiority over NLR, PLR, or PNI. Future adequately powered studies should compare their discrimination, calibration, and incremental prognostic value within the same cohort.

Higher GINI was associated with adverse outcomes in univariable and exploratory threshold-based analyses. However, its independent association was not robust in the core penalized models, the joint MMI–GINI model, or the multiple-imputation analysis. The very broad bootstrap interval around the GINI cutoff further limits threshold interpretation. GINI should therefore be regarded as hypothesis-generating in this cohort rather than an independently validated prognostic marker.

The GINI threshold remained unstable, with a 95% bootstrap interval of 20.104–180.985 around the selected value of 139.67. This wide interval indicates that substantially different thresholds were selected across resamples. It may reflect the skewed GINI distribution, limited event count, and smaller complete-case sample. The threshold should therefore be interpreted as exploratory and cohort-specific, while continuous modeling remains primary and external validation is required.

In the present study, HUAC was evaluated as a measure of masseter muscle radiodensity and an exploratory imaging indicator of myosteatosis, rather than as a validated diagnostic criterion. The number of studies investigating the prognostic significance of muscle radiodensity as a marker of myosteatosis in patients with head and neck cancer remains limited. In most available studies, muscle radiodensity has been assessed at the C3 or L3 vertebral level and regarded as an indicator of overall muscle quality. For example, Ordonez et al. demonstrated that skeletal muscle radiodensity measured at the C3 vertebral level was significantly associated with OS and local control in patients with head and neck cancer [28]. Similarly, another study involving patients with head and neck cancer treated with curative radiotherapy reported that myosteatosis identified at the L3 vertebral level was an independent prognostic factor for OS [29]. Bardoscia et al. also reported that low muscle radiodensity assessed at the L3 vertebral level was an adverse prognostic factor for OS in patients with head and neck cancer [30].

HUAC showed poor three-year discrimination and no statistically significant association in the proportional-hazards penalized models. Its covariate-specific Schoenfeld tests indicated non-proportionality. In adjusted extended Cox analyses, the hazard associated with lower HUAC increased over time, with no clear adverse association early and larger estimates later in follow-up. The 36-month piecewise analysis supported this pattern, but the very wide late OS confidence interval and the small numbers of events after 36 months preclude a definitive claim. This result should therefore be considered hypothesis-generating. Because only voxels within −29 to +150 HU were included in the attenuation calculations, HUAC represents the radiodensity of the thresholded muscle compartment. Excluding very low-attenuation voxels may limit the characterization of fatty infiltration and affect comparability with measurements obtained without this restriction. No sensitivity analysis using unthresholded attenuation values was performed; therefore, the influence of this restriction on the HUAC estimates and their prognostic associations could not be quantified.

In the core-adjusted joint model, lower MMI was associated with OS and PFS, whereas GINI did not show a statistically significant independent association. Additional adjustment for BMI and concurrent systemic therapy attenuated the MMI estimates, although their direction remained consistent. Thus, the strength of the MMI association depended on the adjustment set. Concurrent systemic therapy was allocated according to clinical indication, and its adverse univariable association with survival should not be interpreted as evidence of treatment-related harm. Because BMI and systemic therapy were added together, the attenuation cannot be attributed to either variable alone. These findings do not establish independently complementary prognostic information from MMI and GINI.

MMI and GINI can be obtained from routinely available pretreatment data without additional imaging or invasive procedures. They may identify patients requiring closer nutritional, functional, or inflammatory assessment; however, the present study demonstrates prognostic associations only and does not establish benefit from marker-guided interventions.

Potential clinical applications remain exploratory. Any use for treatment selection, rehabilitation, nutritional intervention, or surveillance requires prospective validation, standardized measurement procedures, and externally validated thresholds.

Limitations include the retrospective single-center design, modest event count, heterogeneous stage and treatment selection, and residual confounding. The predominance of male patients (136 of 146; 93.2%) may also limit the generalizability of the findings to female patients. Residual confounding by treatment indication, systemic agent, and radiotherapy technique cannot be excluded; adjustment for receipt of concurrent systemic therapy does not fully capture regimen-specific differences or unmeasured determinants of treatment selection. Radiotherapy technique was reported descriptively but was not included in the core event-conscious models because only 15 patients received 3D-CRT. All patients completed radiotherapy and total elapsed treatment duration was available, but dates and reasons for individual unscheduled interruptions were not separately coded. GINI was missing in 30 patients, and these patients differed substantially from complete cases; multiple imputation did not confirm an independent GINI association. Although the final masseter contours were agreed upon by consensus, independent blinded repeat measurements were not performed. Consequently, interobserver and intraobserver reproducibility remained unquantified for both MMI and HUAC. Consensus review does not replace a formal reproducibility assessment. In addition, the influence of the −29 to +150 HU restriction could not be quantified because no sensitivity analysis using unthresholded attenuation values was performed. The thresholds were derived and evaluated in the same cohort. Bootstrap internal validation quantified optimism in cutoff-based discrimination but did not externally validate the thresholds or remove selection-related optimism from the Kaplan–Meier group estimates and log-rank comparisons. The GINI threshold was particularly unstable. HUAC also showed an univariable nonlinearity signal, and its late time-varying estimates were based on only 6 OS and 9 PFS events after 36 months; these findings should therefore be interpreted cautiously and require external validation. Functional muscle measures, weight loss, and longitudinal biomarker changes were unavailable. These limitations require cautious interpretation.

Missing GINI data may have introduced clinically meaningful selection bias. Compared with patients with calculable GINI, those without calculable GINI had earlier-stage, predominantly glottic disease, received no concurrent systemic therapy, and accounted for only 2 of the 30 deaths and 8 of the 48 PFS events. Consequently, the complete-case analysis preferentially represented a higher-risk subgroup and may have overestimated the apparent adverse prognostic association of GINI. Multiple imputation attenuated the GINI estimates and did not confirm an independent association with either OS or PFS. However, multiple imputation relies on the missing-at-random assumption and cannot fully account for selection bias related to unmeasured determinants of missingness. The GINI findings should therefore be considered hypothesis-generating.

## 5. Conclusions

To our knowledge, this is the first study to evaluate MMI, HUAC, and GINI within the same cohort of patients with laryngeal squamous cell carcinoma treated with definitive radiotherapy. Lower pretreatment MMI showed the most consistent association with poorer survival, although this association weakened after additional adjustment. GINI was associated with poorer survival in initial analyses, but its independent prognostic value was not consistently supported after accounting for clinical factors and missing data. Lower HUAC showed a possible association with poorer outcomes later in follow-up; however, this finding was based on few events and should be interpreted cautiously. These findings and the cohort-specific MMI and GINI cutoffs require validation in independent cohorts before clinical use.

## Figures and Tables

**Table 1:** Baseline patient, tumor, and treatment characteristics.

Characteristic	All Patients (*n* = 146)
Age group, *n* (%)	
<65 years	87 (59.6)
≥65 years	59 (40.4)
Sex, *n* (%)	
Female	10 (6.8)
Male	136 (93.2)
BMI group, *n* (%)	
<25 kg/m^2^	75 (51.4)
≥25 kg/m^2^	71 (48.6)
ECOG performance status, *n* (%)	
0	64 (43.8)
1	71 (48.6)
2	11 (7.6)
Primary tumor site, *n* (%)	
Supraglottic	23 (15.8)
Glottic	104 (71.2)
Transglottic	19 (13.0)
T category, *n* (%)	
T1	61 (41.8)
T2	35 (24.0)
T3	26 (17.8)
T4a	17 (11.6)
T4b	7 (4.8)
N category, *n* (%)	
N0	104 (71.2)
N1	16 (11.0)
N2	25 (17.1)
N3	1 (0.7)
Overall stage, *n* (%)	
I	57 (39.0)
II	25 (17.1)
III	25 (17.1)
IVA	31 (21.2)
IVB	8 (5.5)
Disease extent, *n* (%)	
Early stage	82 (56.2)
Locally advanced	64 (43.8)
Concurrent systemic therapy, *n* (%)	
No	77 (52.7)
Yes	69 (47.3)
Concurrent regimen among treated patients, *n* (%)	
Cisplatin	57 (82.6)
Cetuximab	12 (17.4)
Smoking exposure, pack-years, median (IQR)	40 (25–50)
Radiotherapy technique, *n* (%)	
IMRT	131 (89.7)
3D-CRT	15 (10.3)
Total RT dose, Gy, median (IQR)	65.25 (63.00–70.00)
Fraction size, Gy: 1.80/2.00/2.12/2.25	1/76/6/63
Elapsed RT duration, days, median (IQR); range	48 (42–51); 37–59
MMI, cm^2^/m^2^, median (IQR)	2.97 (2.55–3.42)
HUAC, HU, median (IQR)	62.14 (54.29–69.36)
GINI, median (IQR), *n* = 116	40.92 (11.80–130.08)
CRP, mg/L, median (IQR)	3.30 (1.37–9.95)
Albumin, g/L, median (IQR), *n* = 116	43.70 (40.96–45.86)
Monocytes, ×10^9^/L, median (IQR)	0.68 (0.51–0.82)
Platelets, ×10^9^/L, median (IQR)	246.5 (202.5–292.5)
Neutrophils, ×10^9^/L, median (IQR)	4.57 (3.69–6.04)
Lymphocytes, ×10^9^/L, median (IQR)	2.12 (1.69–2.63)

Data are presented as *n* (%) or median (IQR), unless otherwise specified. Fraction-size values are reported as patient counts in the order shown. Percentages for concurrent systemic regimens are calculated among the 69 patients receiving concurrent systemic therapy. GINI and albumin summaries are based on 116 patients; the other reported variables are based on 146 patients. BMI, body mass index; ECOG, Eastern Cooperative Oncology Group; IQR, interquartile range; RT, radiotherapy; IMRT, intensity-modulated radiotherapy; 3D-CRT, three-dimensional conformal radiotherapy; MMI, masseter muscle index; HUAC, Hounsfield unit average calculation; HU, Hounsfield units; GINI, Global Immune–Nutrition–Inflammation Index; CRP, C-reactive protein.

**Table 2:** Exploratory three-year time-dependent ROC analysis for overall survival.

Marker	*n*	Cutoff	AUC 95% Bootstrap CI	Sensitivity	Specificity	Youden	95% Bootstrap Cutoff Interval	Optimism-Corrected C-Index
MMI	146	2.830	0.716 (0.592–0.833)	74.6%	68.2%	0.428	2.433–3.027	0.654
GINI	116	139.67	0.786 (0.666–0.892)	64.4%	87.9%	0.523	20.104–180.985	0.704
HUAC	146	50.08	0.485 (0.344–0.621)	22.2%	85.2%	0.074	34.022–91.844	0.493

Abbreviations: AUC, area under the curve; CI, confidence interval; MMI, masseter muscle index; HUAC, Hounsfield unit average calculation; GINI, Global Immune–Nutrition–Inflammation Index; ROC, receiver operating characteristic; OS, overall survival; HU, Hounsfield units. Cutoffs were derived for three-year OS from radiotherapy initiation. Adverse groups were defined by MMI ≤ 2.830 cm^2^/m^2^, GINI ≥ 139.67, and HUAC ≤ 50.08 HU. HUAC was not retained for threshold-based survival visualization. Bootstrap intervals and optimism correction used 1000 patient-level resamples. The HUAC cutoff interval is based on 986 resamples with a finite informative cutoff; 14 resamples did not yield one. C-indices describe cutoff-based grouping and are not validation estimates for the multivariable continuous-marker models.

**Table 3:** Survival outcomes according to masseter muscle index.

Outcome	Group	*n*	Median Months	2-Year Survival % 95% CI	3-Year Survival % 95% CI	Log-Rank *p*
OS	MMI > 2.830	84	Not reached	94.0 (86.1–97.4)	92.5 (84.0–96.6)	<0.001
OS	MMI ≤ 2.830	62	78.9	75.2 (62.3–84.3)	68.9 (55.1–79.3)	
PFS	MMI > 2.830	84	Not reached	88.1 (79.0–93.4)	81.1 (70.5–88.2)	<0.001
PFS	MMI ≤ 2.830	62	56.6	66.0 (52.7–76.3)	59.5 (45.6–70.9)	

Abbreviations: MMI, masseter muscle index; OS, overall survival; PFS, progression-free survival; CI, confidence interval. The log-rank *p*-value compares the two MMI groups. MMI is expressed in cm^2^/m^2^. Survival times were measured from radiotherapy initiation. Confidence intervals use Greenwood’s variance with a log-log transformation and are conditional on the cohort-derived threshold. Group comparisons are exploratory.

**Table 4:** Survival outcomes according to the Global Immune–Nutrition–Inflammation Index.

Outcome	Group	*n*	Median Months	2-Year Survival % 95% CI	3-Year Survival % 95% CI	Log-Rank *p*
OS	GINI < 139.67	88	Not reached	93.1 (85.4–96.9)	90.2 (81.2–95.0)	<0.001
OS	GINI ≥ 139.67	28	27.7	53.3 (33.5–69.7)	45.1 (26.0–62.5)	
PFS	GINI < 139.67	88	Not reached	86.3 (77.2–92.0)	81.9 (71.6–88.7)	<0.001
PFS	GINI ≥ 139.67	28	13.4	46.4 (27.6–63.3)	38.0 (20.2–55.7)	

Abbreviations: GINI, Global Immune–Nutrition–Inflammation Index; OS, overall survival; PFS, progression-free survival; CI, confidence interval. The log-rank *p*-value compares the two GINI groups. Survival times were measured from radiotherapy initiation. Confidence intervals use Greenwood’s variance with a log-log transformation and are conditional on the cohort-derived threshold. Group comparisons are exploratory.

**Table 5:** Univariable Cox regression analyses for overall survival and progression-free survival in the full MMI cohort (*n* = 146).

Variable	OS HR 95% CI	*p*	PFS HR 95% CI	*p*
MMI per 1-SD decrease	2.30 (1.48–3.60)	<0.001	2.14 (1.53–3.01)	<0.001
HUAC per 1-SD decrease	1.14 (0.79–1.66)	0.484	1.15 (0.86–1.55)	0.338
Age ≥ 65 vs. <65 years	1.85 (0.90–3.80)	0.093	1.30 (0.74–2.30)	0.362
Male vs. female	0.54 (0.16–1.80)	0.314	0.88 (0.27–2.87)	0.837
BMI ≥ 25 vs. <25 kg/m^2^	0.28 (0.12–0.65)	0.003	0.38 (0.21–0.70)	0.002
ECOG PS ≥ 2 vs. 0–1	8.06 (3.47–18.72)	<0.001	4.23 (1.94–9.20)	<0.001
Non-glottic vs. glottic primary	5.26 (2.52–11.00)	<0.001	3.34 (1.89–5.92)	<0.001
T3–4 vs. T1–2	7.07 (3.13–15.97)	<0.001	4.13 (2.30–7.43)	<0.001
N-positive vs. N0	5.93 (2.77–12.73)	<0.001	2.91 (1.62–5.20)	<0.001
Stage III–IV vs. I–II	9.58 (3.61–25.41)	<0.001	3.83 (2.08–7.07)	<0.001
Concurrent systemic therapy: yes vs. no	7.67 (2.92–20.15)	<0.001	3.01 (1.65–5.52)	<0.001

Abbreviations: OS, overall survival; PFS, progression-free survival; HR, hazard ratio; CI, confidence interval; MMI, masseter muscle index; HUAC, Hounsfield unit average calculation; BMI, body mass index; ECOG PS, Eastern Cooperative Oncology Group performance status; SD, standard deviation. OS and PFS were measured from radiotherapy initiation. The linear HUAC term summarizes an exploratory constant-effect model; time-varying and nonlinearity analyses are reported separately.

**Table 6:** Univariable Cox regression analyses for overall survival and progression-free survival in the GINI subset (*n* = 116).

Variable	OS HR 95% CI	*p*	PFS HR 95% CI	*p*
GINI per doubling	1.42 (1.23–1.64)	<0.001	1.24 (1.10–1.40)	<0.001
Age ≥ 65 vs. <65 years	1.54 (0.73–3.25)	0.256	1.13 (0.61–2.11)	0.697
Male vs. female	0.61 (0.18–2.03)	0.419	0.85 (0.26–2.77)	0.781
BMI ≥ 25 vs. <25 kg/m^2^	0.31 (0.13–0.74)	0.008	0.38 (0.19–0.74)	0.005
ECOG PS ≥ 2 vs. 0–1	6.52 (2.78–15.25)	<0.001	3.99 (1.80–8.86)	<0.001
Non-glottic vs. glottic primary	4.93 (2.25–10.78)	<0.001	3.21 (1.71–6.02)	<0.001
T3–4 vs. T1–2	6.85 (2.74–17.12)	<0.001	4.31 (2.18–8.52)	<0.001
N-positive vs. N0	4.79 (2.15–10.68)	<0.001	2.88 (1.53–5.42)	0.001
Stage III–IV vs. I–II	11.19 (3.28–38.14)	<0.001	4.24 (1.99–9.01)	<0.001
Concurrent systemic therapy: yes vs. no	7.65 (2.30–25.46)	<0.001	3.63 (1.66–7.91)	0.001

Abbreviations: OS, overall survival; PFS, progression-free survival; HR, hazard ratio; CI, confidence interval; GINI, Global Immune–Nutrition–Inflammation Index; BMI, body mass index; ECOG PS, Eastern Cooperative Oncology Group performance status. OS and PFS were measured from radiotherapy initiation. GINI was log2-transformed, and its hazard ratios (HRs) are expressed per doubling.

**Table 7:** Event-conscious Firth penalized Cox regression and sensitivity analyses.

A. Primary marker-specific models adjusted for age, ECOG PS, primary site, T category, and N status.				
**Primary model marker**	**OS aHR (95% CI)**	***p***	**PFS aHR (95% CI)**	***p***
MMI, per 1-SD decrease	1.92 (1.21–3.05)	0.002	1.91 (1.35–2.71)	<0.001
HUAC, per 1-SD decrease	0.98 (0.65–1.49)	0.929	1.02 (0.74–1.41)	0.890
GINI, per doubling	1.19 (0.99–1.41)	0.050	1.08 (0.93–1.24)	0.309
B. Joint MMI–GINI model with the same core confounders.				
**Joint MMI–GINI model**	**OS aHR (95% CI)**	***p***	**PFS aHR (95% CI)**	***p***
MMI, per 1-SD decrease	1.81 (1.15–2.86)	0.004	1.78 (1.23–2.58)	<0.001
GINI, per doubling	1.19 (0.99–1.42)	0.061	1.07 (0.92–1.24)	0.369
C. Expanded and multiple-imputation sensitivity analyses.				
**Sensitivity analysis marker**	**OS aHR (95% CI)**	***p***	**PFS aHR (95% CI)**	***p***
MMI, expanded single-marker model	1.69 (0.96–2.97)	0.061	1.74 (1.15–2.64)	0.007
GINI, expanded single-marker model	1.18 (0.99–1.41)	0.062	1.08 (0.93–1.25)	0.337
MMI, expanded joint model	1.64 (0.91–2.95)	0.086	1.56 (0.98–2.46)	0.051
GINI, expanded joint model	1.17 (0.98–1.40)	0.083	1.07 (0.92–1.24)	0.395
GINI, multiple imputation	1.15 (0.97–1.37)	0.112	1.03 (0.90–1.18)	0.647

Abbreviations: OS, overall survival; PFS, progression-free survival; aHR, adjusted hazard ratio; HR, hazard ratio; CI, confidence interval; SD, standard deviation; MMI, masseter muscle index; HUAC, Hounsfield unit average calculation; GINI, Global Immune–Nutrition–Inflammation Index; BMI, body mass index; ECOG PS, Eastern Cooperative Oncology Group performance status. All estimates were obtained using Firth penalized Cox regression, with survival measured from radiotherapy initiation. Core adjustment included continuous age, ECOG PS ≥ 2, non-glottic primary site, T3–4 category, and N-positive disease. Expanded models additionally included continuous BMI and concurrent systemic therapy (yes/no). MMI and HUAC effects are expressed per 1-SD decrease, and log2-transformed GINI effects per doubling. Single-marker MMI and HUAC models included 146 patients, with 30 deaths and 48 PFS events. Complete-case GINI and joint MMI–GINI models included 116 patients, with 28 deaths and 40 PFS events. The multiple-imputation analysis included all 146 patients in each of 50 completed datasets; missing pretreatment albumin values were imputed, GINI was recalculated and expanded-model estimates were pooled using Rubin’s rules. The constant HUAC estimates in panel A should be interpreted alongside the non-proportional-hazards analyses.

**Table 8:** Adjusted piecewise extended Cox analysis of HUAC non-proportionality.

Endpoint	Time Interval	Events	HR per 1-SD Decrease in HUAC (95% CI)	*p*	Period-Interaction *p*
OS	0–36 months	24	0.67 (0.43–1.07)	0.092	0.001
OS	>36 months	6	12.21 (2.16–69.06)	0.005	
PFS	0–36 months	39	0.82 (0.58–1.18)	0.289	0.013
PFS	>36 months	9	2.58 (1.11–5.97)	0.027	

Abbreviations: OS, overall survival; PFS, progression-free survival; HR, hazard ratio; CI, confidence interval; SD, standard deviation; HUAC, Hounsfield unit average calculation; ECOG, Eastern Cooperative Oncology Group. Hazard ratios (HRs) are expressed per 1-SD decrease in HUAC and are adjusted for age, ECOG performance status, primary site, T category, and N status. Time intervals are measured from radiotherapy initiation. The period-interaction *p*-value tests whether the HUAC association differs between the 0–36-month and >36-month periods. Late-period estimates are exploratory and imprecise because only 6 OS events and 9 PFS events occurred after 36 months.

## Data Availability

The study data are held by the Akdeniz University Faculty of Medicine and cannot be shared without permission. Researchers seeking access to confidential data should contact the Akdeniz University Corporate Data Access/Ethics Board: etik@akdeniz.edu.tr.

## Supplementary Materials

The following supporting information can be downloaded at: https://www.mdpi.com/article/10.3390/cancers18182981/s1, Table S1: Comparison of patients with and without calculable GINI; Table S2: Nonlinearity assessments using restricted cubic splines.
