# Supplementary material for: Prognostic Value of Masseter Muscle Index, Masseter Radiodensity, and the Global Immune-Nutrition-Inflammation Index in Laryngeal Carcinoma Treated with Definitive Radiotherapy
Source: Cancers (Basel). 2026 Sep 15;18(18):2981. doi: 10.3390/cancers18182981 (PMC13605917; doi:10.3390/cancers18182981)
Supplement: Supplementary file 1 [file cancers-18-02981-s001.zip › cancers-4550533-supplementary.pdf]

Supplementary Tables

Table S1. Comparison of patients with and without calculable GINI.

Characteristic	GINI available (n=116)	GINI missing (n=30)	p
Age, years, median (IQR)	63 (57–70)	62 (52–64)	0.090
MMI, median (IQR)	2.88 (2.51–3.41)	3.20 (2.80–3.59)	0.080
Non-glottic primary, n (%)	40 (34.5)	2 (6.7)	0.003
T3–4, n (%)	49 (42.2)	1 (3.3)	<0.001
N-positive, n (%)	42 (36.2)	0	<0.001
Stage III–IV, n (%)	63 (54.3)	1 (3.3)	<0.001
Concurrent systemic therapy, n (%)	69 (59.5)	0	<0.001
Deaths, n (%)	28 (24.1)	2 (6.7)	0.042
PFS events, n (%)	40 (34.5)	8 (26.7)	0.515
Median reverse-KM follow-up, months	47.2	49.4	—

Continuous variables are presented as median (IQR) and categorical variables as n (%), with percentages calculated within each group. Between-group comparisons used the Mann–Whitney U test for continuous variables and Fisher's exact test for categorical variables. Median follow-up was estimated using the reverse Kaplan–Meier method from radiotherapy initiation; no formal between-group test was performed for follow-up duration. GINI, Global Immune-Nutrition-Inflammation Index; MMI, masseter muscle index, expressed in cm^2/m^2; IQR, interquartile range; PFS, progression-free survival; KM, Kaplan–Meier.

Table S2. Nonlinearity assessments using restricted cubic splines.

Analysis	OS p for nonlinearity	PFS p for nonlinearity
MMI univariable RCS	0.708	0.948
Log2 GINI univariable RCS	0.452	0.501
HUAC univariable RCS	0.054	0.039
HUAC core-adjusted RCS	0.232	0.099
Nonlinear HUAC terms added to the adjusted extended Cox model	0.957	0.387

Abbreviations: RCS, restricted cubic spline; OS, overall survival; PFS, progression-free survival; MMI, masseter muscle index; HUAC, Hounsfield unit average calculation; GINI, Global Immune-Nutrition-Inflammation Index; ECOG, Eastern Cooperative Oncology Group. RCS models used knots at the 10th, 50th, and 90th percentiles. Likelihood-ratio tests used one degree of freedom for the ordinary Cox comparisons and two for adding nonlinear HUAC and nonlinear HUAC-by-log-time terms to the extended model. Core adjustment included age, ECOG performance status, primary site, T category, and N status.
